# Desafios e Oportunidades para o Desenvolvimento da Pesquisa em Educação Médica

**DOI:** 10.36660/abc.20220434

**Published:** 2022-11-09

**Authors:** Sílvia Mamede

**Affiliations:** 1 Institute of Medical Education Research Rotterdam Erasmus Medical Centre Rotterdam Holanda Institute of Medical Education Research Rotterdam , Erasmus Medical Centre , Rotterdam – Holanda

**Keywords:** Educação médica/tendências, Pesquisa Biomédica/tendências, Projetos de Pesquisa, Pesquisa Empírica, Coleta de Dados, Promoção da Pesquisa

O ano era 1747. Médico de bordo do Salisbury, Dr. James Lindt, consternado com o elevado número de mortes por escorbuto entre os marinheiros, planejou e conduziu um estudo comparando diferentes abordagens terapêuticas. Descrito em seu “Tratado sobre escorbuto”, publicado em Edimburgo em 1753, o estudo é considerado o primeiro ensaio clínico controlado da era moderna. ^1^ Mas a história vai muito mais longe. Obviamente sem os requisitos de um ensaio clínico controlado, o experimento conduzido no reinado de Nabucodonosor nos anos 500 AC na Babilônia, é citado como o primeiro registro de um estudo médico que guiou uma decisão de saúde pública. Uma dieta “herbívora” foi autorizada quando, contrariando o que achava o rei, mostrou mais benefícios do que sua preferida alternativa “carnívora”. ^1^

O ano era 1926. É lançado o *Journal of the Association of American Medical Colleges* (hoje *Academic Medicine* ), descrito à época por Fred Zapffe, seu editor, como “a única publicação científica do seu tipo no mundo – um periódico dedicado à educação médica e pedagogia”. Três décadas depois, surgiram os primeiros departamentos de educação médica em universidades americanas, onde muitos veem as origens da pesquisa na área. ^2^ Diante da longa história da pesquisa clínica, a pesquisa em educação médica pode ser vista como recém-nascida. Nos seus poucos anos de vida, seu desenvolvimento tem sido notável. O número de periódicos em educação médica multiplicou-se. O *Science Citation Index* traz 19 e a lista cresce a cada ano. Cresceu substancialmente o número de submissões de artigos científicos a esses periódicos. Por exemplo, nos primeiros 5 anos após sua fundação em 1996, *Advances in Health Sciences Education* recebeu um total de 78 submissões. Só no ano de 2019, o número de submissões foi de 750. ^2^

Há também avanços mais difíceis de quantificar. Parece haver uma mudança de visão em curso. Tempos atrás – e, na verdade, ainda hoje em muitos locais – as decisões educacionais nas escolas médicas, por exemplo quanto à adoção de um método de ensino, eram tomadas com base na opinião de lideranças que detinham mais poder de persuasão, muitas vezes sob a influência de modismos ou posições políticas. Não se falava em evidências. Preponderava a visão de que o senso comum bastava para orientar essas decisões. O passar dos anos e a evolução da pesquisa em educação médica vêm deixando claro que as coisas não são assim. Ideias que pareciam razoáveis ao senso comum e foram adotadas, às vezes em larga escala e para decisões tão importantes quanto exames de certificação, foram abandonadas subsequentemente porque não sobreviveram ao teste da pesquisa empírica. ^3^ Isso é um bom sinal. O abandono de ideias que vêm a mostrar não ter suporte empírico ou a mudança do foco da pesquisa ao longo do tempo são sinais de vida da produção científica no campo. É preciso que evidências se acumulem para que isso aconteça. Falo de evidências aqui no sentido amplo, como um acúmulo de informações empíricas sobre um determinado tópico, e não simplesmente uma “prova” de que uma intervenção “funcionou”. E uma mudança de visão no sentido de se pressupor que, assim como as decisões clínicas, as decisões educacionais precisam de uma base empírica, nesse sentido amplo, ainda que incipiente, abre portas para o desenvolvimento da pesquisa em educação.

Como promover esse desenvolvimento é um tema recorrente de discussão nos periódicos na área na literatura internacional. Como seria de esperar, há diferentes perspectivas, diferentes olhares sobre o problema. ^4 - 6^ A discussão a seguir é em parte orientada por essa literatura, mas representa uma posição pessoal sobre condições que me parecem mais importantes para promover o progresso científico em nosso campo e algumas ideias sobre como elas poderiam ser viabilizadas.

Uma primeira condição crítica para progredir diz respeito ao propósito e ao tipo de pesquisa que é preciso priorizar. Universidades com departamentos de pesquisa em educação médica consolidados destacam-se nas revisões da produtividade científica. ^7^ Esses departamentos contam com pesquisadores e estudantes de doutorado dedicados ao campo da educação médica, muitos oriundos de outras áreas além da medicina, o que permite articular teorias, modelos e métodos de pesquisa próprios de diferentes disciplinas. ^3 , 8^ São departamentos com uma estrutura de suporte à pesquisa e longa tradição de produção científica. Mas tais departamentos são, acredito, a exceção e não a regra. Grande parte da pesquisa em educação médica é conduzida por professores de ciências básicas ou clínicas motivados pelo interesse em educação e/ou em produção científica para progressão acadêmica. Esses professores têm um treinamento formal em pesquisa em educação limitado, quando o têm, um domínio restrito da literatura na área e pouco tempo disponível para investir em suprir essas limitações. A tendência então é realizar estudos pontuais, que acabam em si mesmos. Um professor que introduziu uma nova forma de ensinar um determinado tópico, por exemplo, quer investigar se o novo método “funcionou”. Tudo isso é muito válido, mas é preciso que essa investigação parta do estudo da literatura existente, tenha uma base teórica, esteja inserida numa estrutura conceitual. Isso é fundamental não só para evitar o desperdício de esforços ocorrido, por exemplo, quando se repete o que já foi feito, como para fazer avançar o conhecimento no campo da educação médica. Descobertas que tenham um potencial de produzir mudanças raramente são fruto de um estudo isolado. É preciso uma série de estudos relacionados entre si, que “conversem” com estudos anteriores e construam sobre eles para que se possa avançar na compreensão de um determinado tema. É preciso articular estudos observacionais (por exemplo, a descrição de uma nova intervenção educacional) e estudos que visam testar hipóteses e modelos ou entender como e por que eles funcionam (ou não) para ampliar nosso entendimento do processo de ensino-aprendizagem e direcionar não só a prática, mas a pesquisa em educação. ^3 , 4 , 8^ Essas séries de estudos são sempre resultado do esforço coletivo de diversos grupos e centros ao longo dos tempos. Sempre vai haver questões específicas locais que vale a pena investigar, mas visualizar como este estudo se insere nesse esforço coletivo é essencial para fazer com que ele de fato compense o esforço.

Uma segunda condição essencial é o compromisso com a qualidade metodológica da pesquisa em educação médica. E evito me referir à “melhoria”, porque acho, como muitos, que há pesquisa de elevada qualidade na área. ^9 , 10^ No entanto, são frequentes críticas à qualidade metodológica da pesquisa, usualmente baseadas no pressuposto de que ela utiliza métodos que seriam “inferiores” àqueles utilizados na pesquisa clínica. ^5 , 11^ Por exemplo, se ensaios clínicos controlados randomizados são considerados o padrão ouro na pesquisa para avaliação de uma intervenção terapêutica, então deveríamos estar conduzindo ensaios similares para avaliar a efetividade de um novo curso ou um novo programa. Essa posição, a meu ver, não reconhece que a pesquisa em educação médica tem características próprias que a diferenciam da pesquisa clínica. ^6 , 9 , 10^ Enquanto há, por exemplo, uma segurança razoável quanto ao uso da medicação (ou do placebo) em um ensaio clínico, se (ou quanto, ou como) o “tratamento” foi administrado ao estudante é algo basicamente impossível de controlar. Não se pode estandardizar as “doses” de um curso. Cada um deles consiste, na verdade, em vários elementos, conduzidos por professores diversos, cada um deles com suas características e capacidade próprias. Não é à toa que tais experimentos de grande porte para avaliar currículos inteiros, já foram denominados, numa paródia da sigla em inglês RCT, “ *Results Confounded and Trivial* ”. ^12^ A complexidade inerente ao processo e, consequentemente, à pesquisa educacional, não implica que a pesquisa experimental não tem lugar na educação médica. Na verdade, tem um lugar crucial. O conhecimento que temos hoje em muitas áreas da educação médica foi produzido ao longo dos anos pelo acúmulo de resultados de estudos experimentais de pequeno porte, construídos sobre uma base teórica sobre aquele tema específico, muito bem controlados, conduzidos usualmente em condições laboratoriais, replicados várias vezes para uma variação sistemática dos fatores envolvidos. ^2 - 4^ O que é fundamental, acredito, é entender que alta qualidade metodológica não significa aderir a um determinado tipo de estudo, mas sim buscar métodos que melhor possibilitem examinar o fenômeno em pauta e assumir o compromisso com o máximo rigor em seu uso. É muito provável que a investigação de um fenômeno complexo como costumam ser os fenômenos educacionais demande uma combinação de diferentes métodos de pesquisa, muitas vezes próprios de diferentes disciplinas. Sejam quais forem o desenho e método de pesquisa apropriados, precisamos encontrar maneiras de assegurar que eles obedeçam aos padrões mais elevados para pô-los em prática.

Orientar os esforços para conduzir esse tipo de pesquisa – uma pesquisa fundamentada em uma base teórica e orientada para sua ampliação e de elevada qualidade metodológica - é visto por muitos como crucial para o desenvolvimento científico do campo. Não é fácil viabilizar esse tipo de pesquisa. É preciso domínio substancial da literatura sobre um determinado tema para se apropriar de estruturas conceituais e identificar lacunas, questões que demandam investigar. É preciso dominar os desenhos e métodos de pesquisa que permitem abordar tais questões. É provável que apenas uns poucos dentre aqueles que se interessam pela pesquisa em educação médica nas nossas faculdades venham a optar por dedicar o tempo e o esforço que seriam necessários para adquirirem tal domínio. A articulação de duas linhas de ação pode, acredito, ajudar. A primeira seria dar a esses interessados em se dedicarem de forma mais intensa à pesquisa em educação médica a oportunidade de desenvolverem a expertise necessária, formando, ao longo do tempo, um núcleo de pesquisadores que possa orientar, apoiar e assegurar a qualidade da pesquisa no seu contexto. A segunda seria ampliar o suporte disponível para o grupo bem maior de docentes que tem interesse em realizar pesquisa em educação, mas não como principal objeto de sua atividade profissional.

Relatos de experiências internacionais sugerem algumas iniciativas que podem ajudar nos dois sentidos. ^3^ Uma palavra-chave parece ser “cooperação”. A articulação com universidades que possuem programas acreditados de mestrado e doutorado em educação médica, com uma produção científica reconhecida, é fundamental para viabilizar a formação de professores que venham a se dedicar à pesquisa na área como atividade principal. Um treinamento formal mais avançado é necessário para formar uma “massa crítica de cientistas” que a experiência mostra ser um fator crítico para o desenvolvimento do campo. ^13^ Várias universidades atualmente oferecem programas de boa qualidade, inclusive em formato híbrido, que poderiam potencialmente ser viabilizados utilizando os esquemas usuais de suporte à pós-graduação. Em nível local, a articulação com outras faculdades e centros na própria universidade pode ajudar a abrir a possibilidade de atrair profissionais de outras disciplinas, por exemplo, das faculdades de ciências sociais, com conhecimento e experiência, inclusive em relação a métodos de pesquisa, carentes na própria instituição. ^8^ A cooperação entre diversas instituições tanto em nível local como internacional pode ajudar também a ampliar a estrutura de suporte à pesquisa, agregando esforços e recursos, por exemplo, para a oferta de cursos de menor duração para um amplo grupo de docentes. Para finalizar, a iniciativa louvável deste suplemento dos Arquivos Brasileiros de Cardiologia (ABC Cardiol) chama atenção para o papel que as sociedades médicas podem exercer nesse processo. A credibilidade e a influência na comunidade profissional e na sociedade as credenciam para isso. Iniciativas bem sucedidas existem em que uma sociedade médica foi um parceiro importante no esforço para alavancar a capacidade de pesquisa na área. ^14^ Defender a importância da pesquisa em educação médica, fomentar a discussão de estratégias para seu desenvolvimento e articular a cooperação entre diversas instituições que, tanto no contexto nacional como internacional, podem ampliar a capacidade de pesquisa existente e podem certamente contribuir para promover o progresso científico na área.
