# Supplementary material for: Desafios e Oportunidades para o Desenvolvimento da Pesquisa em Educação Médica
Source: Arq Bras Cardiol. 2022 Nov 9;119(5 Suppl 1):1–3. [Article in Portuguese] doi: 10.36660/abc.20220434 (PMC9750198; doi:10.36660/abc.20220434)
Supplement: Translation

# Translation: Challenges and Opportunities for the Development of Medical Education Research

**Authors:** Sílvia Mamede

**Affiliations:** 1 Netherlands Institute of Medical Education Research Rotterdam, Erasmus Medical Centre, Rotterdam – Netherlands

**Keywords:** Education, Medical/trends, Biomedical Research/trends, Research Design, Empirical Research, Data Collection, Research Promotion

The year was 1747. The doctor on board the Salisbury, Dr. James Lindt, upset with the high number of deaths by scurvy among the mariners, planned and conducted a study comparing different therapeutic approaches. Described in his “Treatise of the Scurvy”, published in Edinburgh in 1753, his study is considered to be the first controlled clinical trial in the modern era.^1^ But the history goes even further. Clearly, without the requirements of a controlled clinical trial, the experiment, conducted during the reign of King Nebuchadnezzar in the years of 500 BC in Babylonia, is cited as the first record of a medical study that guided a public health decision. A “herbivorous” diet was authorized when, contradicting what the King believed, it showed more benefits than the preferred “carnivorous” alternative.^1^

The year was 1926. The *Journal of the Association of American Medical Colleges (known today as Academic Medicine) was launched described* at that time by Fred Zapffe, its editor, as “the only scientific publication of its type in the world – a journal dedicated to medical education and pedagogy.” Three decades later, the first departments of medical education emerged in American universities, which many believe to be the origins of medical research.^2^ In view of the long history of clinical research, medical education research can be seen as newborn. In its few years of life, its development has been quite remarkable. The number of journals on medical education have multiplied. The *Science Citation Index* offers 19 publications, and the list continues to grow each year. The number of scientific article submissions to these journals has also increased significantly. For example, in the first 5 years since its foundation in 1996, *Advances in Health Sciences Education* had received a total of 78 submissions. In 2019 alone, the number of submissions reached 750.^2^

There have also been advances that are more difficult to quantify. There appears to be a change in mentality taking place. Some time ago – and, in fact, still today in many places – the educational decisions in medical schools, as regards the adoption of a teaching method, for example, were made based on the opinion of department heads who had greater power of persuasion, often under the influence of fads or political positions. No one spoke of evidence. The view that prevailed was that common sense was enough to guide decisions. Over the years, and with the evolution in medical education research, it has become clear that this is not true. Ideas that seemed reasonable and were adopted, often in large scale and for decisions as important as certification exams, were subsequently abandoned because they were unable to survive the test of empirical evidence.^3^ This is a good sign. The abandonment of ideas that are proven to have no empirical support, or the change in focus of research over time, are clear signs of the life of scientific production in the field. For this to occur, evidence has to “accumulate”. I speak of evidence here in the broader sense, an accumulation of empirical evidence about a specific topic, and not merely “proof” that a given intervention “worked”. It represents a change in mentality in the sense of assuming that, much like the clinical decision, the educational decisions also require an empirical basis, in this broader sense. Even if in its incipient stage, it opens doors to the development of research in education.

How to promote this development is a recurring theme of debate in international medical journals. As expected, there are different perspectives, different views on the problem.^4 - 6^ The following discussion is partly guided by this literature, but it represents a personal position on the conditions that seem to me to be more important to promoting scientific progress in our field, along with some ideas about how this can be made possible.

One first critical condition necessary to advance on the issue concerns the purpose and the type of research to which it is necessary to give priority. Universities with departments dedicated to medical education research have stood out in reviews of scientific production.^7^ These departments have researchers and doctoral students dedicated to the medical education field, many from areas outside of medicine, which gives space for the exchange of theories, models, and methods from different disciplines.^3 , 8^ These are departments with a strong research support structure and a long tradition of scientific production. But this type of department is clearly the exception and not the rule. Most medical education research is conducted by professors from basic sciences or from clinics who are motivated by their interests in education and/or scientific production for academic promotion. These professors have limited formal training in education research, when they have any; a limited acquaintance with of the literature in the area; and little time available to invest in overcoming these limitations. The tendency is, therefore, to conduct occasional studies about their own work. A professor who introduced a new way to teach a specific topic, for example, may wish to investigate if this new method “works”. All of this is quite valid, but it is important for this type of investigation to begin with the study of the existing literature, to have a theoretical basis, and to be inserted in a conceptual structure. This is essential not only to avoid wasting time and effort, for example, when a person repeats what has already been done, but also to advance knowledge in the medical education field. Discoveries that have the potential to produce changes are rarely the result of an isolated study. What is important is a series of interconnected studies that “talk” with previous studies and build upon them so that progress can be made in understanding a given theme. It is important to connect observational studies (for example, the description of a new educational intervention) and studies that seek to test hypotheses and models or understand how and why they work (or not) in order to expand our understanding of the teaching-learning process and direct not only the practice, but also research in education.^3 , 4 , 8^ These series of studies are always the result of a collective effort from a diversity of groups and centers over time. There will always be specific local questions that are worth investigating; however, comprehending how this study fits within the collective effort is essential to making it truly worth the effort.

A second essential condition is that of commitment to the methodological quality of medical education research. And here I avoid referring to “improvements”, because I think, as many do, that there is high-quality research being conducted in the area.^9 , 10^ However, criticism of the research methodological quality is common, usually based on the assumption that it uses methods that would be “inferior” to those used in clinical research.^5 , 11^ For instance, if randomized controlled trials are considered the gold standard in research for the evaluation of a therapeutic intervention, then we should be conducting similar trials to evaluate the effectiveness of a new course or a new program. This position, from my point of view, does not recognize that medical education research has its own characteristics that differentiate it from clinical research.^6 , 9 , 10^ While there is, for example, a reasonable degree of certainty about the use of a medicine (or a placebo) in a clinical trial, if (or how much, or how) the “treatment” was administered to the student is something basically impossible to control. The “doses” of the course cannot be standardized. Each course consists, in fact, of various elements, conducted by a wide range of teachers, each with his/her own characteristics and skills. It is not by accident that this type of large-scale experiment to evaluate full curricula have become known, in the parody of the acronym in English, as RCT - “ *Results Confounded and Trivial* ”.^12^ The complexity inherent to the process and, consequently, to educational research, does not imply that experimental research has no place in medical education. In fact, it plays a crucial role. The knowledge we have today in many areas of medical education was produced over the years through the accumulation of small-scale experimental studies, built upon a theoretical foundation about that specific theme – highly controlled, usually conducted under laboratory conditions, and replicated many times to reach a systematic variation of the factors involved.^2 - 4^ What is of utmost importance, I believe, is understanding that a high methodological quality does not mean adhering to a specific type of study, but rather to search for methods that are more appropriate to examine the phenomenon in question and to assume the responsibility with their careful application. It is highly probable that the investigation of a complex phenomenon, as educational phenomena commonly are, demands a combination of different research methods, often brought from different disciplines. Whatever the most appropriate study design and method may be, we need to ensure that they comply with the highest of standards when putting them into practice.

Directing our efforts to conducting this type of research – research founded on a theoretical basis and oriented towards its expansion and with high level methodological quality – is seen by many as crucial to scientific development in the field. It is not easy to make this type of research possible. It requires a substantial mastery of the literature on a given theme so as to take advantage of the conceptual structures and identify gaps, questions that require investigation. It is important to master the study designs and methods that allow one to treat such questions. It is likely that only a few of those who are interested in medical education research within our universities will actually opt to dedicate the time and effort needed to acquire such mastery. Combining two lines of action can, I believe, help. The first would be to give researchers interested in dedicating themselves intensely to medical education research the opportunity to develop the necessary expertise, constructing, over time, a research group that can guide, support, and ensure the quality of the research in its context. The second would be to expand the support available to a much larger groups of faculty members who are interested in conducting research in the field of education, but not as the main focus of their professional work.

Reports of international experiences suggest some initiatives that can help in both directions.^3^ A key word seems to be “cooperation”. Connecting with universities that have accredited Masters and Ph.D. programs in medical education, with a well-recognized scientific production, is essential in order to enable the education of teachers who choose to dedicate themselves to research in the area as their main professional activity. A formal, more advanced training is necessary to create a “critical mass of scientists”, whose experience has proven to be a critical factor in the development of the field.^13^ Many universities currently offer high-quality programs, even in hybrid formats, which can potentially be made possible using the existing schemes for post-graduation support. At the local level, the interaction with other colleges and research centers in the university itself can help to open the door to the possibility of attracting other professionals from other disciplines, such as from the social sciences, with knowledge and experience regarding research methods that may be lacking within one’s own faculty.^8^ The cooperation between several institutions, both at the local and international levels, can also help to expand the research support structure, adding efforts and resources, including shorter-term courses for a larger group of faculty members. To conclude, this laudable initiative of this supplement of the journal *Arquivos Brasileiros de Cardiologia* (ABC Cardiol) calls attention to the role that medical societies can play in this process. The credibility and influence in the professional community and in society itself qualify these societies for this. Highly successful initiatives already exist in which a medical society became an important partner in the effort to boost research skills in the field.^14^ Defending the importance of medical education research, fostering the debate on strategies for its development, and formulating cooperation among a wide range of institutions, in both the national and the international contexts, can help expand existing research skills and clearly contribute to promoting scientific progress in the field.
